# Antifungal activity of selected volatile essential oils against *Penicillium* sp.

**DOI:** 10.1515/biol-2020-0045

**Published:** 2020-07-19

**Authors:** Soňa Felšöciová, Nenad Vukovic, Paweł Jeżowski, Miroslava Kačániová

**Affiliations:** Department of Microbiology, Faculty of Biotechnology and Food Science, Slovak University of Agriculture in Nitra, Tr. A. Hlinku 2, 949 76 Nitra, Slovakia; Department of Chemistry, Faculty of Science, University of Kragujevac, P.O. Box 12, Kragujevac, Serbia; Institute of Chemistry and Technical Electrochemistry, Faculty of Chemical Technology, Poznan University of Technology, Berdychowo 4, 60-965 Poznań, Poland; Department of Fruit Sciences, Viticulture and Enology, Faculty of Horticulture and Landscape Engineering, Slovak University of Agriculture, Tr. A. Hlinku 2, 94976 Nitra, Slovakia; Department of Bioenergy, Technology and Microbiology, Institute of Food Technology and Nutrition, University of Rzeszow, Cwiklinskiej 1, 35601 Rzeszow, Poland

**Keywords:** antimicrobial activity, disc diffusion method, concentration of plant essential oils, fungi isolated from grape

## Abstract

Phytopathogenic fungi have been responsible for considerable economic losses in vineyards, and therefore, more attention should be paid to the development and implementation of preventative treatment that is environmentally friendly. The aim of this study was to evaluate the antifungal activity of ten essential oils (EOs) (viz. *Lavandula angustifolia* Mill., *Carum carvi* L., *Pinus mugo* var. *pumilio*, *Mentha piperita* L., *Foeniculum vulgare* L., *Pinus sylvestris* L., *Satureja hortensis* L., *Origanum vulgare* L., *Pimpinella anisum* L. and *Rosmarinus officinalis* L.). For the antifungal activity evaluation against *Penicillium brevicompactum*, *P. citrinum*, *P. crustosum*, *P. expansum*, *P. funiculosum*, *P. glabrum*, *P. chrysogenum*, *P. oxalicum*, *P. polonicum* and *Talaromyces purpurogenus* a disc diffusion method was used. The ten EOs exhibited different antifungal properties. Three tested EOs (*Carum carvi* L., *Satureja hortensis* L. and *Pimpinella anisum* L.) at concentrations of 0.75, 0.50, 0.25 and 0.125 µL/mL showed antifungal activity, inhibiting the mycelial growth. The *Origanum vulgare* L. EOs exhibited a lower level of inhibition. Overall, *Lavandula angustifolia* Mill., *Pinus mugo* var. *pumilio*, *Mentha piperita* L., *Foeniculum vulgare* L., *Pinus sylvestris* L., *Satureja hortensis* L., *Pimpinella anisum* L. and *Rosmarinus officinalis* L. were effective as fungicidal agents but their efficiency varied between the strains of fungi. *Carum carvi* L. showed strong antifungal activity against all tested strains at both full strength and reduced concentrations. These EOs could be considered as potential sources of antifungal compounds for treating plant fungal diseases.

## 1 Introduction

Many *Penicillium* species are soil fungi, while others find their habitat in decaying vegetables, seeds or fruits, which are ecological niches that play a role in the food rotting process. For example *P. expansum* causes decay of oranges in the citrus industry or rot in grapes [1]. Moreover, these species are known as the major producers of patulin and many other toxic metabolites such as citrinin, roquefortine C or chaetoglobosins among others [2]. Growth of *Penicillium* in food products is entirely undesirable, especially as many *Penicillium* species produce mycotoxins and volatile secondary metabolites that are regarded as health hazards and off-flavors [3].

Medicinal plants are sometimes used by different ethnic groups as a natural source of substances used as a cure for diseases of both humans and domestic animals [1]. Some of the plant natural products can have various biological activities such as anti-inflammatory, anticarcinogenic, anti-atherosclerotic, antibacterial, antifungal, antiviral, antimutagenic and antiallergic activities [4,5,6,7,8,9,10,11,12]. The antimicrobial activities of plant extracts have many applications, including raw and processed food preservation, as pharmaceuticals, alternative medicines and natural therapies [13,14].

Essential oils (EOs) are secondary metabolites produced by vascular plants, mostly different species of the labiate family *Lamiaceae*, *Apiaceae* and *Asteraceae*, and other families such as *Rutaceae*, *Lauraceae* and *Myrtaceae* [15].

EOs can be composed of more than 60 components. Phenolic compounds are responsible for the antimicrobial activity of EOs [16]. The effect of EOs on molds can be followed at the macromorphological level, as well as at the cellular level. Some of the macromorphological changes are the lack of sporulation or pigmentation, change in the number of conidia, increased branching of hyphae or change in their size. It has been proposed that some of the mentioned changes are related to the oil activities on enzymatic reactions of cell wall synthesis, which affect mold growth and morphogenesis, and also cause the pulling back of the cytoplasm in hyphae, whereby mycelium death occurs [17]. EOs can inhibit the synthesis of DNA, RNA, proteins and polysaccharides in fungi and bacterial cells, where they can cause changes, similar to the mechanism of antibiotic activity [18,19].

Search for alternative antifungal substances shows the possible use of EOs and extracts for food protection from mycotoxigenic molds and their toxic products [20]. An important role of EOs in nature is protection of plants by acting as antifungal agents. The hypothesis is how different volatile EOs in different concentrations influence different plant fungal strains of *Penicillium* sp. The aim of this study is to present the antifungal properties of ten EOs against ten *Penicillium* strains isolated from plants.

## 2 Materials and methods

### 2.1 Plant EOs

The EOs used in this study were commercial samples from Calendula a.s., Nová Ľubovňa, Slovakia (*Lavandula angustifolia* Mill., *Carum carvi* L., *Pinus mugo* var. *pumilio*, *Mentha piperita* L., *Foeniculum vulgare* L., *Pinus sylvestris* L., *Satureja hortensis* L., *Origanum vulgare* L., *Pimpinella anisum* L. and *Rosmarinus officinalis* L.). All samples were stored at 4°C in a dark glass flask until analysis. Pure EOs were dissolved in DMSO (dimethylsulfoxide; Penta, Czech Republic) at different concentrations. The 0.75 µL/mL (mass per volume) solutions thus prepared were diluted to 0.375, 0.1875 and 0.09375 µL/mL and immediately analyzed.

### 2.2 Fungal strains and media

The selected plant fungal strains *P. brevicompactum*, *P. citrinum*, *P. crustosum*, *P. expansum*, *P. funiculosum*, *P. glabrum*, *P. chrysogenum*, *P. oxalicum*, *P. polonicum* and *Talaromyces purpurogenus* (previously called *Penicillium purpurogenum*) were obtained from the fungal culture collection bank at the Department of Microbiology, Slovak University of Agriculture in Nitra. Fungal strains were maintained on Czapek yeast extract agar (CYA, HiMedia, Bombay, India), and the cultures were stored at −21°C. Genus *Penicillium* that was 7 days old was identified to the species level based on macroscopic and microscopic characteristics according to the manuals of Pitt [21], Samson and Frisvad [22] and Samson et al. [23]. After microscopic identification, strains of fungi were confirmed with a MALDI-TOF MS Biotyper (Bruker Daltonics, Bremen, Germany).

### 2.3 Disc diffusion method

Seven-day-old cultures grown on agar plates (CYA) were used for the preparation of the mold conidia suspensions. Conidia suspensions were prepared in a sterile saline solution. The turbidity of the suspension was adjusted with a spectrophotometer – densilameter II (Erba-Lachema, Brno, Czech Republic) at 530 nm to obtain a final concentration that matches that of a 0.5 McFarland standard. Briefly, 100 µL of spore suspension (0.5 McF) was spread thoroughly all over the surface of Sabouraud dextrose agar (SDA; Hi-Media Laboratory, India) plates. The plates were dried in an air-dry stiller at 60°C until evaporation of residual water. Sterile paper discs (6 mm in diameter; Oxoid, Cambridge, UK) were impregnated with 20 µL of EO containing the test compound at a desired concentration (0.75, 0.50, 0.25 and 0.125 µL/mL/disc) and deposited on the agar surface. The test for antifungal properties of EOs was repeated three times, for each microorganism and each concentration. The Petri dishes were incubated at 25 ± 1°C, for 24 h in a thermostat (Friocell, MMM Medcenter Einrichtungen GmbH, Germany). After 24 h of incubation period, the antifungal agent diffused into the agar and inhibited the germination and growth of the tested microorganism. The diameters of inhibition growth zones were measured as semidiameter (in millimeters). Pure DMSO was used as control for each tested fungus [24].

### 2.4 Chemical composition of EOs

The chemical composition of EOs (*Lavandula angustifolia* Mill., *Carum carvi* L., *Pinus mugo* var. *pumilio*, *Mentha piperita* L., *Foeniculum vulgare* L., *Pinus sylvestris* L., *Satureja hortensis* L., *Origanum vulgare* L., *Pimpinella anisum* L. and *Rosmarinus officinalis* L.) was determined by gas chromatography/mass spectrometry and is reported elsewhere [25].

### 2.5 Statistical analysis

For calculating the average values and standard deviation of the obtained data, MS Excel (Microsoft Office Professional Plus 2010, Microsoft, USA) was used. The reported values are the mean values from the tests repeated three times.

## 3 Results and discussion

The chemical analyses by CG/MS revealed that the main constituents of *Lavandula angustifolia* Mill. were linalool (39.31%) and linalyl acetate (37.68%), for *Carum carvi* L., carvone (69.54%) and limonene (21.12%); for *Pinus mugo* var. *pumilio*, α-pinene (21.26%); for *Mentha piperita* L., menthol (28.56%) and menthone (27.39%); for *Foeniculum vulgare* L., anethole (24.98%); for *Pinus sylvestris* L., α-pinene (26.15%), camphene (15.51%) and bornyl acetate (14.59%); for *Satureja hortensis* L., carvacrol (41.23%) and γ-terpinene (32.11%); for *Origanum vulgare* L., carvacrol (43.26%); for *Pimpinella anisum* L., anethole (63.25%); and for *Rosmarinus officinalis* L., 1,4-cineole (21.26%), α-pinene (15.65%) and *p*-cymene (13.28%) [24].

The antifungal properties of different EOs against the growth of *Penicillium brevicompactum* on SDA are presented in Table 1. The EOs of *Lavandula angustifolia* Mill. showed strong antifungal activity against *Penicillium brevicompactum* with a zone of inhibition ranging from 10.33 ± 3.67 to 19.67 ± 0.82 mm. The EOs of *Pinus sylvestris* L., *Satureja hortensis* L., *Origanum vulgare* L. and *Rosmarinus officinalis* L. exhibited the least antifungal activity with an inhibition zone from 0.17 ± 0.41 to 3.17 ± 0.41 mm against *P. brevicompactum*. The EO concentration of 0.75 µL/mL showed the most effective inhibition of the growth of *Penicillium brevicompactum*, followed by 0.50, 0.25 and 0.125 µL/mL concentrations. According to Felšöciová et al. [24], the EOs of *Pimpinella anisum* L. exhibited the highest antifungal activity against *P. brevicompactum* at all observed concentrations (0.75, 0.375, 0.1875 and 0.09375 µL/mL) after incubation for 24 h compared to the control sample. However, the EOs of *Pinus mugo* var. *pumilio* exhibited the least antifungal activity in the concentration range from 0.25 ± 0.50 to 3.00 ± 2.16 mm against all ten tested oils. The reported results do not correspond with our observations. In our case, *Pinus mugo* var. *pumilio* exhibited strong antifungal properties and *Pimpinella anisum* L. showed a moderate activity limiting the growth of the mentioned fungus. According to D’Auria et al. [26], lavender oil showed both fungistatic and fungicidal activities against *Candida albicans* strains. Markovic et al. [27] studied the antifungal activity of thymol and carvacrol on *Aspergillus* spp. and *Penicillium* spp., and they found that both thymol and carvacrol have potential antifungal activity, but the susceptibility of *Aspergillus* spp. was more than that of *Penicillium* spp.

**Table 1:** Measured sizes of inhibition zones (in mm) for various EOs at different concentrations (mean ± SD) against *Penicillium brevicompactum*

EO	Concentration of EO (µL/mL)			
	0.75	0.50	0.25	0.125
1. *Lavandula angustifolia* Mill.	19.67 ± 0.82	15.67 ± 2.94	13.50 ± 1.38	10.33 ± 3.67
2. *Carum carvi* L.	4.50 ± 1.22	4.17 ± 0.75	3.50 ± 0.84	3.00 ± 0.63
3. *Pinus mugo* var. *pumilio*	14.33 ± 1.75	9.33 ± 3.50	3.50 ± 0.55	1.33 ± 0.52
4. *Mentha piperita* L.	4.50 ± 0.55	4.00 ± 0.89	2.50 ± 0.55	1.67 ± 0.82
5. *Foeniculum vulgare* L.	3.33 ± 1.03	2.50 ± 0.55	1.50 ± 0.55	1.00 ± 0.00
6. *Pinus sylvestris* L.	3.17 ± 0.41	2.17 ± 0.75	1.00 ± 0.63	0.17 ± 0.41
7. *Satureja hortensis* L.	2.67 ± 0.52	2.00 ± 0.00	1.33 ± 0.52	0.67 ± 0.52
8. *Origanum vulgare* L.	2.83 ± 0.41	1.67 ± 0.52	0.83 ± 0.41	0.25 ± 0.42
9. *Pimpinella anisum* L.	6.17 ± 0.41	5.50 ± 0.55	5.00 ± 0,63	2.67 ± 0.52
10. *Rosmarinus officinalis* L.	2.17 ± 1.17	1.67 ± 0.82	1.67 ± 0.74	1.33 ± 0.52
DMSO (negative control)	NE	NE	NE	NE

NE – non-inhibitory effect.

The antifungal activities of EOs against *Penicillium citrinum* are shown in Table 2. The best antifungal activity was found for the EO of *Pinus sylvestris* L. (from 1.17 ± 0.75 to 9.50 ± 1.41 mm) and the lowest was for *Pimpinella anisum* L., *Rosmarinus officinalis* L. and *Origanum vulgare* L. with a zone of inhibition from 1.00 ± 0.00 to 2.17 ± 0.98 mm. A non-inhibitory effect was observed for *Mentha piperita* L. and *Foeniculum vulgare* L. at all concentrations tested. Felšöciová et al. [24] reported that the EO of *Pimpinella anisum* L. was very active against *Penicillium citrinum*, but the inhibition zones were not measurable, and also the EO of *Origanum vulgare* L. had a strong antifungal activity with an inhibition zone ranging from 2.75 ± 0.96 to 12.0 ± 1.83 mm (at concentrations 0.75, 0.375, 0.1875 and 0.09375), which is in contrast to our observations. The EOs of *Pinus sylvestris* L., *Mentha piperita* L. and *Rosmarinus officinalis* L. had the lowest activities (from 0.75 ± 0.50 to 3.25 ± 1.26 mm). These findings are in agreement with previous results except for *Pinus sylvestris* L., for which the inhibition zone ranged from 1.17 ± 0.75 to 9.50 ± 1.41 mm. Scalas et al. [28] evaluated the antifungal activity of *Origanum vulgare* (oregano), *Pinus sylvestris* L. (pine) and *Thymus vulgaris* (thyme red) EOs against *Cryptococcus neoformans* clinical strains. All EOs displayed an antifungal activity against the *C. neoformans* isolate, and the order from the most to the least effective EO is as follows: oregano > pine > thyme EOs. Guynot et al. [29] reported that the volatile fraction of five tested EOs (cinnamon leaf, clove, bay, lemongrass and thyme) had potential antifungal activity against the more common fungi causing spoilage of bakery products (*Eurotium amstelodami*, *E. repens*, *E. rubrum*, *Aspergillus flavus*, *A. niger* and *Penicillium corylophilum*). The same effect was observed by Rodríguez et al. [30], that is, the clove EO totally inhibited all of the tested isolates including two *Penicillium* species (*P. nalgiovense* and *P. roqueforti*). Lis-Balchin and Deans [15] reported that strong antimicrobial activity could be correlated with EOs containing high percentages of monoterpenes, eugenol, cinnamic aldehyde and thymol.

**Table 2:** Measured sizes of inhibition zones (in mm) for various EOs at different concentrations (mean ± SD) against *Penicillium citrinum*

EO	Concentration of EO (µL/mL)			
	0.75	0.50	0.25	0.125
1. *Lavandula angustifolia* Mill.	4.17 ± 0.98	2.67 ± 0.82	2.00 ± 1.26	1.17 ± 0.75
2. *Carum carvi* L.	3.67 ± 0.82	3.17 ± 0.75	1.83 ± 0.75	1.50 ± 0.55
3. *Pinus mugo* var. *pumilio*	3.50 ± 1.38	3.67 ± 1.63	1.50 ± 1.64	0.50 ± 1.26
4. *Mentha piperita* L.	NE	NE	NE	NE
5. *Foeniculum vulgare* L.	NE	NE	NE	NE
6. *Pinus sylvestris* L.	9.50 ± 1.41	5.00 ± 1.73	3.00 ± 1.41	1.17 ± 0.75
7. *Satureja hortensis* L.	5.83 ± 1.03	5.33 ± 0.82	2.67 ± 0.82	1.33 ± 0.52
8. *Origanum vulgare* L.	1.67 ± 0.52	1.17 ± 0.41	1.00 ± 0.00	1.00 ± 0.00
9. *Pimpinella anisum* L.	2.17 ± 0.98	2.00 ± 1.10	2.00 ± 1.10	1.50 ± 0.98
10. *Rosmarinus officinalis* L.	1.83 ± 0.98	2.17 ± 1.47	1.50 ± 0.55	1.00 ± 1.26
DMSO (negative control)	NE	NE	NE	NE

NE – non-inhibitory effect.

EOs which showed the strongest antifungal activity against *Penicillium crustosum* are *Carum carvi* L., *Foeniculum vulgare* L. and *Satureja hortensis* L. (from 6.17 ± 1.33 to 6.67 ± 3.14 mm) at a concentration of 0.75 µL/mL. Other EOs showed a moderate impact on the growth of the mentioned fungus (Table 3). In our previous study, the best antifungal activity against *Penicillium crustosum* was shown by *Pimpinella anisum* L., and a strong inhibition effect was also exhibited at a concentration of 0.75 µL/mL by *Chamomilla recutita* L. and *Thymus vulgaris* L. [24]. *Origanum vulgare* L. EOs showed an excellent antifungal activity against the tested fungus *P. crustosum* for which the zone of inhibition ranges from 3.00 ± 0.82 mm at a concentration of 0.09375 µL/mL to 12.50 ± 1.73 mm at the highest concentration (0.75 µL/mL). A moderate antifungal effect was shown by the oils of *Carum carvi* L. and *Satureja hortensis* L. Similar studies have shown the antifungal activity of some EOs including the study of Zyani et al. [31], who reported the important activity of *Origanum compactum*, *Eugenia caryophyllata* and *Ocimum basilicum* EOs against *Penicillium commune*, *Penicillium chrysogenum* and *Penicillium expansum.* Soidrou et al. [32] have found that Comorian EOs isolated from *Piper capense*, *Piper borbonense* and *Vetiveria zizanoides* have a strong fungicidal activity against fungi decaying wood. Several authors have attributed the antifungal activity of EOs to their major phenolic components [33]. Hassan et al. [34] have shown the important antifungal activity of carvacrol against *P. expansum*. The antifungal activity of the same component against *A. niger*, *A. flavus*, *P. citrinum* and *P. chrysogenum* was studied [35].

**Table 3:** Measured sizes of inhibition zones (in mm) for various EOs at different concentrations (mean ± SD) against *Penicillium crustosum*

EO	Concentration of EO (µL/mL)			
	0.75	0.50	0.25	0.125
1. *Lavandula angustifolia* Mill.	4.83 ± 1.17	4.67 ± 1.37	2.58 ± 1.28	1.17 ± 0.41
2. *Carum carvi* L.	6.67 ± 3.14	5.50 ± 2.43	3.67 ± 2.66	1.00 ± 0.00
3. *Pinus mugo* var. *pumilio*	3.00 ± 0.63	1.83 ± 0.41	1.50 ± 0.55	1.17 ± 0.41
4. *Mentha piperita* L.	3.50 ± 0.84	1.83 ± 0.98	1.83 ± 0.41	1.00 ± 0.00
5. *Foeniculum vulgare* L.	6.17 ± 1.33	5.33 ± 1.51	1.33 ± 1.51	0.83 ± 0.98
6. *Pinus sylvestris* L.	5.83 ± 0.75	2.17 ± 0.41	2.00 ± 1.26	1.00 ± 0.00
7. *Satureja hortensis* L.	6.33 ± 3.14	3.50 ± 1.52	2.17 ± 0.41	1.67 ± 0.52
8. *Origanum vulgare* L.	4.17 ± 1.60	3.17 ± 0.41	2.17 ± 0.41	2.00 ± 0.63
9. *Pimpinella anisum* L.	4.83 ± 1.47	2.83 ± 0.98	1.17 ± 0.41	0.50 ± 0.55
10. *Rosmarinus officinalis* L.	3.67 ± 0.82	2.67 ± 0.52	1.83 ± 0.98	0.83 ± 0.98
DMSO (negative control)	NE	NE	NE	NE

NE – non-inhibitory effect.

The antifungal effects of the ten tested EOs against *Penicillium expansum* are presented in Table 4 *. Penicillium expansum* was the most sensitive to the EO of *Mentha piperita* L. at a concentration of 0.75 µL/mL (9.83 ± 2.56 mm). The weakest inhibitory effect was observed for the EOs of *Pinus sylvestris* L. and *Pinus mugo* var. *pumilio*, for which at a concentration of 0.125 µL/mL there was no inhibitory effect. Plavčič et al. [16] presented that the mint EO, in the case of the disc diffusion method, exhibited antifungal activities against eight tested molds. The largest inhibition for the quantity of 0.5 µL was measured against *P. expansum* growth (14.33 ± 0.58 mm), which is similar to our measurements. A higher inhibition effect was noticed also against *P. expansum* growth (15.33 ± 0.58 mm). According to the results of Felšöciová et al. [24], a high antagonistic effect against *P. expansum* was found in *Thymus vulgaris* L. and *Origanum vulgare* L. with an inhibition zone from 3.50 ± 1.25 up to 12.00 ± 1.63 mm, but the best antifungal activity at all concentrations was shown by *Pimpinella anisum* L. and *Chamomilla recutita* L. The activities of EOs of *Pinus sylvestris* L. and *Pinus mugo* var. *pumilio* were measured at all concentrations, but with a low zone of inhibition from 0.25 ± 0.50 to 1.75 ± 0.50 mm, which is similar to our studies. The concentration of oregano EO required to inhibit the growth of *P. expansum* was found to be from 3 to 5%, and the difference in required concentrations might be attributed to the variations in the chemical composition of the oregano EOs used and also the use of different substrates and due to the resisting mode of the fungi against various substances present in EOs [36]. The obtained results in the study demonstrated that three compounds (β-ionone, carvone and 1,8-cineole) have real antifungal potential and they could be used as antifungal agents as well as to significantly reduce (or completely eliminate) the growth of *Penicillium expansum* during the storage of apples [37].

**Table 4:** Measured sizes of inhibition zones (in mm) for various EOs at different concentrations (mean ± SD) against *Penicillium expansum*

EO	Concentration of EO (µL/mL)			
	0.75	0.50	0.25	0.125
1. *Lavandula angustifolia* Mill.	6.50 ± 1.22	3.83 ± 0.75	2. 38 ± 1.17	0.67 ± 0.52
2. *Carum carvi* L.	4.50 ± 2.43	5.00 ± 2.28	3.00 ± 0.63	1.67 ± 0.82
3. *Pinus mugo* var. *pumilio*	1.67 ± 0.82	1.17 ± 0.68	0.33 ± 0.52	NE
4. *Mentha piperita* L.	9.83 ± 2.56	7.17 ± 2.14	3.67 ± 1.37	0.83 ± 0.40
5. *Foeniculum vulgare* L.	3.33 ± 1.51	3.67 ± 2.25	2.00 ± 1.26	0.50 ± 0.55
6. *Pinus sylvestris* L.	1.67 ± 0.82	2.50 ± 1.38	1.67 ± 0.82	0.67 ± 0.52
7. *Satureja hortensis* L.	4.00 ± 1.10	2.50 ± 1.52	1.00 ± 0.00	0.67 ± 0.52
8. *Origanum vulgare* L.	3.83 ± 1.72	2.83 ± 1.72	2.50 ± 1.76	0.50 ± 0.55
9. *Pimpinella anisum* L.	3.50 ± 1.38	4.50 ± 2.17	2.67 ± 0.82	0.33 ± 0.52
10. *Rosmarinus officinalis* L.	3.00 ± 1.26	2.17 ± 1.17	1.50 ± 0.84	1.00 ± 0.89
DMSO (negative control)	NE	NE	NE	NE

NE – non-inhibitory effect.

The highest antifungal activity against *Penicillium funiculosum* was observed for the extracts of *Lavandula angustifolia* Mill. with an inhibition zone from 1.67 ± 0.52 to 4.00 ± 0.63 mm (Table 5). The lowest antifungal activity was measured for the EOs of *Pimpinella anisum* L. and *Origanum vulgare* L., for which at a concentration 0.125 µL/mL there was no inhibitory effect. Its oil (LEO) has antimicrobial, antifungal, antioxidant, anti-inflammatory, antidepressant, sedative, hypnotic, analgesic and anticancer activity [38,39]. Its impact on reducing the amount of *Candida albicans* fungus has been shown in *in vitro* [40] and clinical studies [41]. Motiejūnaite and Peciulyte [42] determined the fungistatic activity of the volatile fraction of pine oil against fungus species: a strong inhibition effect on the growth of *Penicillium funiculosum* and *Trichoderma viride* was reported. The antifungal activity of 15 chemically defined EOs, alone and in mixture, was checked by a microdilution test against isolates of *Penicillium funiculosum*. *Origanum vulgare* yielded the lowest minimal inhibition concentration (MIC) values, followed by *Salvia sclarea*, *Ocimum basilicum* and *Cymbopogon citratus*, while *Citrus paradisi* and *Citrus limon* were not active. All mixtures showed antifungal activity at lower concentration with respect to MIC values of each EO component, when not in combination [43].

**Table 5:** Measured sizes of inhibition zones (in mm) for various EOs at different concentrations (mean ± SD) against *Penicillium funiculosum*

EO	Concentration of EO (µL/mL)			
	0.75	0.50	0.25	0.125
1. *Lavandula angustifolia* Mill.	4.00 ± 0.63	3.67 ± 0.52	2.17 ± 0.75	1.67 ± 0.52
2. *Carum carvi* L.	3.50 ± 0.55	3.00 ± 1.10	1.67 ± 0.82	1.00 ± 0.00
3. *Pinus mugo* var. *pumilio*	2.83 ± 0.75	2.17 ± 0.41	1.33 ± 0.52	1.00 ± 0.00
4. *Mentha piperita* L.	1.17 ± 0.41	2.00 ± 0.00	2.18 ± 0.40	0.17 ± 0.41
5. *Foeniculum vulgare* L.	3.67 ± 0.52	2.50 ± 0.55	1.83 ± 0.41	1.00 ± 0.00
6. *Pinus sylvestris* L.	2.33 ± 0.82	2.00 ± 0.89	1.83 ± 0.98	0.83 ± 0.41
7. *Satureja hortensis* L.	3.33 ± 0.52	3.17 ± 0.41	1.50 ± 0.55	1.50 ± 0.55
8. *Origanum vulgare* L.	1.00 ± 0.00	0.83 ± 0.41	0.83 ± 0.41	NE
9. *Pimpinella anisum* L.	1.17 ± 0.41	1.00 ± 0.00	0.50 ± 0.55	0.50 ± 0.55
10. *Rosmarinus officinalis* L.	3.00 ± 1.10	3.17 ± 1.33	1.83 ± 0.98	1.33 ± 0.52
DMSO (negative control)	NE	NE	NE	NE

NE – non-inhibitory effect.

The screening results of the ten EOs for their activity against the growth of *Penicillium glabrum* are shown in Table 6. The EO of *Lavandula angustifolia* Mill. was very active and the inhibition zone was 15.50 ± 1.38 mm at 0.75 µL/mL concentration. On the other hand, low activity was observed for EOs from *Rosmarinus officinalis* L., *Foeniculum vulgare* L., *Pinus mugo* var. *pumilio*, *Mentha piperita* L., *Pinus sylvestris* L. and *Origanum vulgare* L., which at 0.125 µL/mL concentration showed no zone of inhibition. However, a number of studies report on a strong antifungal activity of basil EO. Dube et al. [44], using the agar plate method, showed that basil oil at a concentration of 1.5 mL/L inhibited completely the growth of 22 species of molds. The study performed by Lis-Balchin et al. [45] points to a strong antifungal effect of an oil that contained estragole as the main component on the growth of *Aspergillus niger*, *A. ochraceus* and *Fusarium culmorum*.

**Table 6:** Measured sizes of inhibition zones (in mm) for various EOs at different concentrations (mean ± SD) against *Penicillium glabrum*

EO	Concentration of EO (µL/mL)			
	0.75	0.50	0.25	0.125
1. *Lavandula angustifolia* Mill.	15.50 ± 1.38	11.83 ± 1.72	4.17 ± 3.60	0.17 ± 0.41
2. *Carum carvi* L.	7.50 ± 0.55	4.50 ± 0.55	2.83 ± 0.41	3.00 ± 0.63
3. *Pinus mugo* var. *pumilio*	0.67 ± 0.82	0.50 ± 0.55	NE	NE
4. *Mentha piperita* L.	0.83 ± 0.98	0.50 ± 0.55	0.17 ± 0.26	NE
5. *Foeniculum vulgare* L.	0.50 ± 0.55	0.50 ± 0.55	NE	NE
6. *Pinus sylvestris* L.	1.00 ± 0.00	0.33 ± 0.52	0.25 ± 0.27	NE
7. *Satureja hortensis* L.	2.17 ± 1.33	1.00 ± 1.10	0.50 ± 0.55	0.50 ± 0.55
8. *Origanum vulgare* L.	1.33 ± 0.52	0.67 ± 0.32	NE	NE
9. *Pimpinella anisum* L.	2.50 ± 0.55	1.67 ± 0.52	1.20 ± 0.50	0.50 ± 0.55
10. *Rosmarinus officinalis* L.	0.33 ± 0.41	NE	NE	NE
DMSO (negative control)	NE	NE	NE	NE

NE – non-inhibitory effect.

As presented in Table 7, the EOs have strong to moderate antimicrobial activities against the *Penicillium chrysogenum* tested. In the present study, *Pimpinella anisum* L., *Satureja hortensis* L. and lastly *Mentha piperita* L. exhibit remarkable antifungal activity against *Penicillium chrysogenum.* The EO of mint (*Mentha piperita* L.) was used for the purpose of antifungal activity testing against eight different fungi by Plavsic et al. [16]. The inhibition zone was not observed only when the smallest quantity of EO was applied (0.5 µL) against *P. expansum* (14.33 ± 0.58 mm). The quantity of 1 µL showed inhibitory activity against all tested molds. When the highest quantity of EO was applied (10 µL), the complete inhibition of *A. alternata* and *A. versicolor* growth occurred. The inhibition zone of other species was in the range from 13.67 mm (*P. chrysogenum*) to 44.67 mm (*P. aurantiogriseum*). Plavsic et al. [16] concluded that the mint EO had the strongest impact on *Eurotium herbariorum*, and the weakest on *P. chrysogenum*. According to Motiejūnaite and Peciulyte [42], *P. chrysogenum* was the least susceptible to pine oil. Slight antifungal activity of pine oil was shown against *Aspergillus niger*, *A. versicolor* and *Stachybotrys chartarum*. The EO from the plant *Satureja hortensis* L. showed different antimicrobial activities against *Aspergillus niger* and *Candida albicans* [46]. *Candida albicans* showed moderate sensitivity to the oil’s activity, and *Aspergillus niger* manifested a strong resistance to this oil. Other studies revealed a new biological activity for *S. hortensis* L., which is the strong inhibition of aflatoxin production by *Aspergillus parasiticus*. Carvacrol and thymol, and the effective constituents of *S. hortensis* L., may be useful in controlling aflatoxin contamination of susceptible crops in the field [47]. Kambiz et al. [48] clearly demonstrate that the alcoholic extract of *S. hortensis* contains compounds possessing antifungal properties. The alcoholic extract of *S. hortensis* showed antifungal activity against phytopathogenic fungi [49] and against food spoilage fungi [50]. Therefore, on the basis of the results in previous studies, *S. hortensis* can be added as a protective agent to various food products [47].

**Table 7:** Measured sizes of inhibition zones (in mm) for various EOs at different concentrations (mean ± SD) against *Penicillium chrysogenum*

EO	Concentration of EO (µL/mL)			
	0.75	0.50	0.25	0.125
1. *Lavandula angustifolia* Mill.	2.50 ± 0.55	1.33 ± 0.52	1.17 ± 0.41	1.00 ± 0.00
2. *Carum carvi* L.	3.83 ± 0.75	2.83 ± 0.75	1.67 ± 0.52	0.83 ± 0.98
3. *Pinus mugo* var. *pumilio*	3.50 ± 0.55	2.83 ± 0.75	1.83 ± 0.75	0.83 ± 0.41
4. *Mentha piperita* L.	5.50 ± 1.52	4.17 ± 1.47	3.17 ± 0.75	0.50 ± 0.55
5. *Foeniculum vulgare* L.	4.00 ± 0.89	5.50 ± 2.17	2.00 ± 1.10	1.33 ± 1.63
6. *Pinus sylvestris* L.	4.00 ± 0.63	3.33 ± 0.82	2.83 ± 0.98	2.17 ± 0.98
7. *Satureja hortensis* L.	6.50 ± 2.07	2.00 ± 0.00	1.67 ± 0.52	0.17 ± 0.41
8. *Origanum vulgare* L.	3.83 ± 0.41	2.33 ± 0.82	1.67 ± 0.52	0.83 ± 0.98
9. *Pimpinella anisum* L.	6.50 ± 5.21	5.83 ± 4.62	4.50 ± 2.05	1.17 ± 0.41
10. *Rosmarinus officinalis* L.	3.50 ± 0.84	4.17 ± 1.72	1.83 ± 0.45	1.00 ± 0.00
DMSO (negative control)	NE	NE	NE	NE

NE – non-inhibitory effect.

The obtained results from Tables 8 and 9 demonstrate that the highest antifungal activities of *Carum carvi* L. and *Rosmarinus officinalis* L. do not differ against *Penicillium oxalicum* and *P. polonicum*. At a concentration of 0.125 µL/mL, *Pinus mugo* var. *pumilio* indicated no zone of inhibition compared to the control sample for both tested species, which is similar to *Lavandula angustifolia* Mill. against *P. oxalicum* and *Pinus sylvestris* L. against *P. polonicum.* The EO of *Origanum vulgare* L. showed no activity at all against the growth of *P. polonicum* for any of the used concentrations. The EO of caraway (*Carum carvi* L.) has a wide application in pharmaceutical and food industries as it possesses antitumor, antiproliferative, antihyperglycemic and antimicrobial activity [51]. The EO of caraway, in the case of the disc diffusion method at concentrations of 0.5, 1, 5 and 10 µL, exhibited antifungal activity against eight tested molds. Inhibitory activity using the smallest quantity (0.5 µL) was recorded against all isolates, and the highest inhibition zone was observed against *P. chrysogenum* (33.67 mm). By using higher concentrations of caraway EO (1 and 5 µL), a greater antifungal effect was observed on all of the tested molds. Total inhibition was noticed against *Eurotium herbariorum* when using the highest quantity of oil (10 µL), while the highest inhibition zone was observed against *A. versicolor* (52 mm), and the lowest against *A. niger* (28 mm). Helal et al. [52] reported that application of 50 µL of the caraway EO, in the agar diffusion method, did not show inhibition zones against *A. flavus*, while in the case of *A. niger*, *Penicillium digitatum* and *P. puberulum*, the inhibition zones were 22, 18 and 27 mm, respectively. Baghlou et al. [53] detected *in vitro* the antifungal effect of *Rosmarinus officinalis* L. (rosemary) EO against 16 fungal strains of *A. niger* contaminating various food products and responsible for invasive fungal infection. The colonies of the 16 tested strains of *A. niger* showed a very weak growth at 0.25% concentration of the EO. From a concentration of 0.50%, they noted complete absence of growth of the 16 tested strains. The *R. officinalis* L. EO also displayed powerful inhibitory and fungicidal activity against specific *Candida* strains [54]. In the disc diffusion assay, Hendel et al. [55] tested the effect of rosemary extracts on the growth of the green mold of citrus, *Penicillium digitatum*, under *in vitro* conditions. The effect was very strong on spore germination, and the diameter of the inhibition zone was estimated to be 14, 20 and 32.5 mm at the concentrations of 15, 20 and 25 µL/mL, respectively, with the lack of sporulation and sparse mycelium compared to the control. These results support the studies on rosemary as a promising source of preservatives. The antifungal activities of ethanolic extracts of *Rosmarinus officinalis* and *Thymus vulgaris* were tested by Centeno et al. [56] against strains of *Aspergillus flavus* and *A. ochraceus*, since these two species are responsible for accumulating mycotoxins that are common contaminants of cereals and grains. These extracts used at low concentrations could have significant potential for the biological control of fungi in food products.

**Table 8:** Measured sizes of inhibition zones (in mm) for various EOs at different concentrations (mean ± SD) against *Penicillium oxalicum*

EO	Concentration of EO (µL/mL)			
	0.75	0.50	0.25	0.125
1. *Lavandula angustifolia* Mill.	1.00 ± 0.00	0.92 ± 0.20	NE	NE
2. *Carum carvi* L.	6.00 ± 3.10	5.33 ± 3.78	2.17 ± 1.47	1.33 ± 0.52
3. *Pinus mugo* var. *pumilio*	1.00 ± 0.00	0.42 ± 0.49	NE	NE
4. *Mentha piperita* L.	2.83 ± 0.41	2.33 ± 0.52	1.17 ± 0.41	0.50 ± 0.55
5. *Foeniculum vulgare* L.	3.83 ± 1.72	1.83 ± 0.98	1.00 ± 1.10	0.83 ± 0.98
6. *Pinus sylvestris* L.	3.67 ± 1.21	2.50 ± 1.52	1.17 ± 0.41	0.50 ± 0.55
7. *Satureja hortensis* L.	2.58 ± 0.49	1.67 ± 0.41	1.17 ± 0.41	0.75 ± 0.27
8. *Origanum vulgare* L.	0.50 ± 0.55	0.50 ± 0.55	0.50 ± 0.55	0.50 ± 0.55
9. *Pimpinella anisum* L.	2.33 ± 2.58	1.83 ± 2.04	1.17 ± 1.33	0.33 ± 0.52
10. *Rosmarinus officinalis* L.	4.67 ± 1.51	3.17 ± 1.33	2.00 ± 1.10	0.50 ± 0.55
DMSO (negative control)	NE	NE	NE	NE

NE – non-inhibitory effect.

**Table 9:** Measured sizes of inhibition zones (in mm) for various EOs at different concentrations (mean ± SD) against *Penicillium polonicum*

EO	Concentration of EO (µL/mL)			
	0.75	0.50	0.25	0.125
1. *Lavandula angustifolia* Mill.	2.67 ± 1.03	1.67 ± 0.82	0.92 ± 0.58	0.33 ± 0.52
2. *Carum carvi* L.	5.00 ± 3.74	3.83 ± 3.13	3.33 ± 3.13	1.50 ± 1.76
3. *Pinus mugo* var. *pumilio*	2.67 ± 0.52	1.67 ± 0.52	1.17 ± 0.41	NE
4. *Mentha piperita* L.	4.67 ± 0.52	3.17 ± 0.41	2.50 ± 0.55	1.00 ± 0.00
5. *Foeniculum vulgare* L.	2.83 ± 0.41	0.92 ± 0.58	0.50 ± 0.55	0.33 ± 0.52
6. *Pinus sylvestris* L.	1.67 ± 0.82	1.00 ± 0.55	0.33 ± 0.52	NE
7. *Satureja hortensis* L.	2.50 ± 0.55	1.67 ± 0.82	1.17 ± 0.75	0.67 ± 0.52
8. *Origanum vulgare* L.	NE	NE	NE	NE
9. *Pimpinella anisum* L.	3.67 ± 0.52	3.17 ± 0.41	3.00 ± 0.00	0.50 ± 0.55
10. *Rosmarinus officinalis* L.	5.67 ± 1.03	3.00 ± 0.63	2.33 ± 1.21	1.67 ± 1.00
DMSO (negative control)	NE	NE	NE	NE

NE – non-inhibitory effect.

The antifungal activities of various EOs against the growth of *Talaromyces purpurogenus* (previously *Penicillium purpurogenum*) are presented in Table 10. The EOs of *Satureja hortensis* L. and *Pinus sylvestris* L. are the most effective against this fungus with a zone of inhibition ranging from 1.00 ± 0.00 to 7.67 ± 4.50 mm. The antagonistic effect was not found at 0.125 µL/mL for the oil of *Origanum vulgare* L. against the tested fungus. The turpentine oil extracted from *Pinus sylvestris* L. showed a significant antifungal effect on fungal plant pathogens *Sclerotinia sclerotiorum*, *Fusarium oxysporum*, *Botrytis cinerea*, *Phytophthora capsici*, *Alternaria solani* and *Pythium* sp., respectively, but it was more active against bacteria and yeast than fungi, and the antimicrobial activity of the oil increased with an increase of oil concentration in the medium [57].

**Table 10:** Measured sizes of inhibition zones (in mm) for various EOs at different concentrations (mean ± SD) against *Talaromyces purpurogenus*

EO	Concentration of EO (µL/mL)			
	0.75	0.50	0.25	0.125
1. *Lavandula angustifolia* Mill.	2.83 ± 1.17	2.50 ± 0.84	2.00 ± 0.00	1.50 ± 0.55
2. *Carum carvi* L.	3.67 ± 1.03	2.67 ± 0.82	1.67 ± 0.52	1.50 ± 0.55
3. *Pinus mugo* var. *pumilio*	2.33 ± 0.52	1.33 ± 0.52	1.33 ± 0.52	1.17 ± 0.41
4. *Mentha piperita* L.	2.17 ± 1.33	1.67 ± 0.82	1.00 ± 0.00	0.67 ± 0.52
5. *Foeniculum vulgare* L.	3.00 ± 0.89	2.50 ± 0.55	1.67 ± 0.82	1.00 ± 0.00
6. *Pinus sylvestris* L.	4.17 ± 1.83	4.67 ± 1.37	2.00 ± 0.89	1.50 ± 0.55
7. *Satureja hortensis* L.	7.67 ± 4.50	2.67 ± 0.82	2.00 ± 0.63	1.00 ± 0.00
8. *Origanum vulgare* L.	3.00 ± 0.00	2.50 ± 0.55	0.83 ± 0.41	NE
9. *Pimpinella anisum* L.	2.17 ± 0.75	1.50 ± 0.55	1.17 ± 0.41	1.00 ± 0.00
10. *Rosmarinus officinalis* L.	2.83 ± 0.75	2.50 ± 0.55	2.50 ± 0.84	2.17 ± 0.82
DMSO (negative control)	NE	NE	NE	NE

NE – non-inhibitory effect.

## 4 Conclusion

The presented EOs obtained from the selected plants showed different antifungal activities against *Penicillium* species, depending on the concentration of the EO used, as well as the type of microorganism. The highest antifungal activity was observed for the *Lavandula angustifolia* Mill. EO against *Penicillium brevicompactum*. The zone of inhibition varied between 19.67 ± 0.82 and 10.33 ± 3.67 mm depending on the concentration of the EO. The plant extract of *Origanum vulgare* L. did not possess any strong antifungal activity. At high doses, all tested oils were active against the tested strains, except *Mentha piperita* L. and *Foeniculum vulgare* L. against *Penicillium citrinum* and *Origanum vulgare* L. against *P. polonicum*. Diluted oils proved to be less effective and some of them were even inactive: *Lavandula angustifolia* Mill., *Pinus mugo* var. *pumilio*, *Mentha piperita* L., *Foeniculum vulgare* L., *Pinus sylvestris* L., *Origanum vulgare* L. and *Rosmarinus officinalis* L.
